# Supplementary material for: Rhein Improves Renal Fibrosis by Restoring Cpt1a-Mediated Fatty Acid Oxidation through SirT1/STAT3/twist1 Pathway
Source: Molecules. 2022 Apr 6;27(7):2344. doi: 10.3390/molecules27072344 (PMC9000220; doi:10.3390/molecules27072344)
Supplement: Supplementary file 1 [file molecules-27-02344-s001.zip › molecules-1595743-SI.pdf]

molecules

MDPI

Rhein Improves Renal Fibrosis by Restoring Cpt1a-Mediated Fatty Acid Oxidation through SirT1/STAT3/Twist1 Pathway

Xianrui Song [1], Zesen Du [1], Zhenqi Yao [1], Xiaoyan Tang [1] and Mian Zhang [1,2,*]

[1] School of Traditional Chinese Medicine, China Pharmaceutical University, Nanjing 211198, China
[2] Mian Zhang, School of Traditional Chinese Medicine, China Pharmaceutical University, No.639 Longmian Road, Nanjing 211198, China
* herbs@cpu.edu.cn; Tel.: +86-2586185137

Supplemental materials

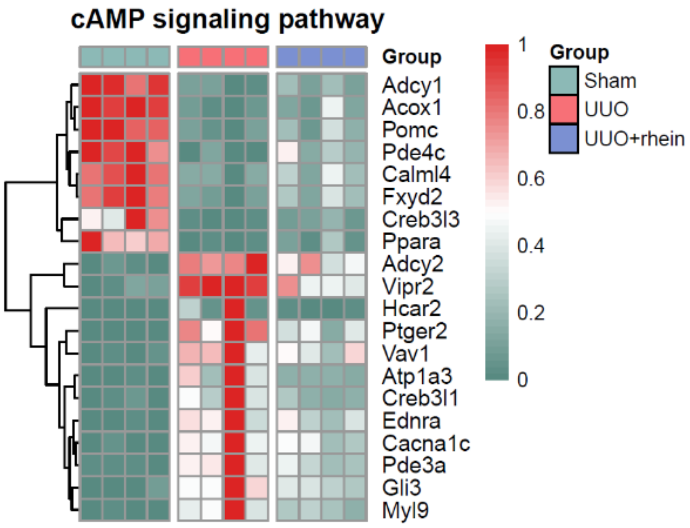

Figure S1. Heatmap of RNA sequencing genes associated with cAMP signaling pathway (*n* = 4).

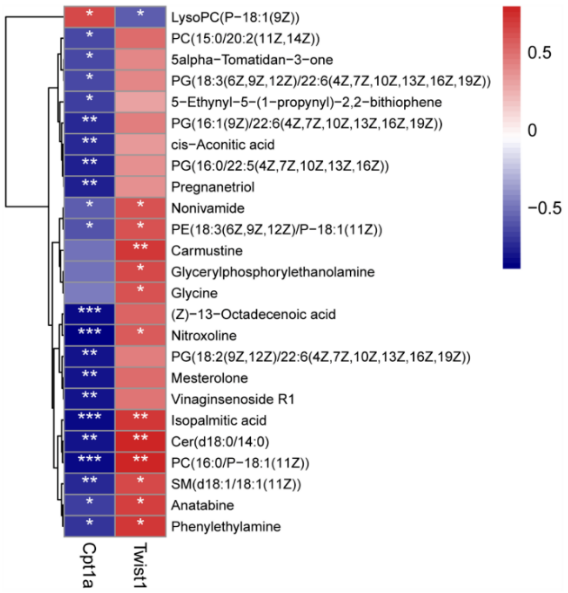

Figure S2. Heatmap of the relationship between Twist1 & Cpt1a genes and differential metabolites. * $p < 0.05$, ** $p < 0.01$, *** $p < 0.001$, gene correlated metabolites.

Table S1. Primers for q- PCR

Primers	Direction	Sequences
Acot1	Forward	5'-ACCGCTTTTGGTTTACA-3'
	Reverse	5'-GATTGAACCCAGGAACTT-3'
Acta2	Forward	5'-CACTACCATGTACCCAGGCA-3'
	Reverse	5'-CATATAGCGGTGGACAATGGA-3'
Col1a1	Forward	5'-CAGGCTGGTGTGATGGGATT-3'
	Reverse	5'-CACCTCGTTCTCCAGCCTTT-3'
Col2a1	Forward	5'-CGCTCAAGTCGCTGAACAACCA-3'
	Reverse	5'-ACCAGTTCTTCCGACGCACAGT-3'
Col3a1	Forward	5'-AGTGGCCATAATGGGGAACG-3'
	Reverse	5'-CAG GGTTTCCATCCCTTCCG-3'
Col4a1	Forward	5'-GTGCGGTTTGTGAAGCACCG-3'
	Reverse	5'-GTTCTTCTCATGCACACTTC-3'
Cpt1a	Forward	5'-ACCCAGTCAGATTCCAACCA-3'
	Reverse	5'-TCAAGAAAGGCACTCCCACT-3'
Cpt1b	Forward	5'-CATGTATCGCCGCAAACTGG-3'
	Reverse	5'-CCTGGGATGCGTGTAGTGTT-3'
Cpt1c	Forward	5'-GGATGGCACTGAAGAGGAAA-3'
	Reverse	5'-TCCTGGAAAAGGCATCTCTC-3'
Cpt2	Forward	5'-GCCCAAACCCCATTTTCTA-3'
	Reverse	5'-TAGGCAGAGGCAGAAGACAGCA-3'
Decr1	Forward	5'-GTACGGCAATGGCTTTATCA-3'
	Reverse	5'-CAATCCCCTCCTGCAACTT-3'
E-cadherin	Forward	5'-AGGTCGGTGCCCGTATTG-3'
	Reverse	5'-TGGTCTTGGGGTCTGTGATG-3'
Fn	Forward	5'-GTTGGTTGCCCTGTTCTGC-3'
	Reverse	5'-GGCTACCTGTGTTTCCCTTTG-3'
Twist1	Forward	5'-CGCACCCAGTCGCTGAACG-3'
	Reverse	5'-CCGCCAGTTTGAGGGTCTGAATC-3
TGF-β	Forward	5'-CATGGAGCTGGTGAAACGGAAG-3'
	Reverse	5'-GACTGGCGAGCCTTAGTTTGGAC-3'
Vimentin	Forward	5'-TCAGACAGGATGTTGACAAT-3'
	Reverse	5'-GACATGCTGTTCCTGAATCT-3'
β-actin	Forward	5'-CCGAGGGCCCACTAAAGG-3'
	Reverse	5'-GCTGTTGAAGTCACAGGAGACAA-3'

Table S2. KEGG analysis of transcriptome data

Description	q-value
Cytokine-cytokine receptor interaction	7.43E−21
Viral protein interaction with cytokine and cytokine receptor	2.92E−14
Cell adhesion molecules	6.10E−13
PI3K-Akt signaling pathway	2.90E−12
Hematopoietic cell lineage	5.95E−11
Chemokine signaling pathway	1.49E−09
Dilated cardiomyopathy	1.81E−09
ECM-receptor interaction	2.37E−09
Osteoclast differentiation	5.60E−09
Hypertrophic cardiomyopathy	1.57E−08

TNF signaling pathway	2.37E−08
MAPK signaling pathway	2.74E−08
Arrhythmogenic right ventricular cardiomyopathy	2.90E−08
Toxoplasmosis	6.04E−08
JAK-STAT signaling pathway	7.69E−08
Amoebiasis	1.05E−07
Complement and coagulation cascades	1.46E−07
Epstein-Barr virus infection	1.82E−07
Human papillomavirus infection	1.86E−07
Focal adhesion	2.93E−07
Rap1 signaling pathway	3.17E−07
Platelet activation	4.43E−07
Human T-cell leukemia virus 1 infection	1.61E−06
NF-kappa B signaling pathway	1.61E−06
Malaria	1.99E−06
Leishmaniasis	2.09E−06
Measles	2.53E−06
Small cell lung cancer	3.69E−06
AGE-RAGE signaling pathway in diabetic complications	3.93E−06
Th17 cell differentiation	3.93E−06
Chagas disease	3.93E−06
Coronavirus disease – COVID-19	7.08E−06
Kaposi sarcoma-associated herpesvirus infection	8.51E−06
Th1 and Th2 cell differentiation	8.51E−06
Ras signaling pathway	1.21E−05
Pertussis	1.21E−05
Viral myocarditis	1.49E−05
Tuberculosis	1.49E−05
Protein digestion and absorption	1.63E−05
Rheumatoid arthritis	2.00E−05
Legionellosis	2.11E−05
Inflammatory bowel disease	2.32E−05
Valine, leucine and isoleucine degradation	2.80E−05
Phagosome	3.75E−05
Human cytomegalovirus infection	3.75E−05
Relaxin signaling pathway	4.06E−05
Yersinia infection	4.82E−05
Salmonella infection	5.62E−05
Leukocyte transendothelial migration	5.62E−05
PD-L1 expression and PD-1 checkpoint pathway in cancer	1.17E−04
Parathyroid hormone synthesis, secretion and action	1.41E−04
Influenza A	1.41E−04
Toll-like receptor signaling pathway	1.48E−04
Primary immunodeficiency	1.60E−04
Glyoxylate and dicarboxylate metabolism	1.70E−04
C-type lectin receptor signaling pathway	2.06E−04
Circadian entrainment	2.39E−04
Transcriptional misregulation in cancer	2.81E−04
Gap junction	3.37E−04
Glycosaminoglycan biosynthesis - chondroitin sulfate / dermatan	3.49E−04

sulfate	
Axon guidance	3.49E−04
T cell receptor signaling pathway	4.68E−04
Tryptophan metabolism	5.49E−04
Hepatitis B	5.61E−04
Hepatitis C	6.71E−04
Lipid and atherosclerosis	6.80E−04
Apoptosis	8.34E−04
Proteoglycans in cancer	8.34E−04
Intestinal immune network for IgA production	9.20E−04
NOD-like receptor signaling pathway	9.25E−04
cAMP signaling pathway	9.52E−04
Cysteine and methionine metabolism	9.77E−04
Regulation of actin cytoskeleton	1.09E−03
Oxytocin signaling pathway	1.18E−03
Prostate cancer	1.18E−03
Staphylococcus aureus infection	1.22E−03
Glycine, serine and threonine metabolism	1.62E−03
Lysine degradation	1.78E−03
Adrenergic signaling in cardiomyocytes	1.96E−03
Fc gamma R-mediated phagocytosis	1.96E−03
Alcoholic liver disease	2.09E−03
Natural killer cell mediated cytotoxicity	2.26E−03
B cell receptor signaling pathway	2.53E−03
Amphetamine addiction	2.82E−03
Viral carcinogenesis	3.18E−03
Cocaine addiction	3.18E−03
Breast cancer	3.22E−03
Cell cycle	5.23E−03
Hepatocellular carcinoma	5.23E−03
Glutamatergic synapse	5.69E−03
Notch signaling pathway	6.45E−03
Insulin secretion	6.87E−03
Cellular senescence	7.09E−03
Phospholipase D signaling pathway	7.17E−03
Morphine addiction	7.18E−03
Cholinergic synapse	8.19E−03
Glioma	1.04E−02
Vascular smooth muscle contraction	1.08E−02
IL-17 signaling pathway	1.09E−02
cGMP-PKG signaling pathway	1.09E−02
Acute myeloid leukemia	1.13E−02
ABC transporters	1.15E−02
Cushing syndrome	1.25E−02
Fluid shear stress and atherosclerosis	1.25E−02
Other types of O-glycan biosynthesis	1.26E−02
Colorectal cancer	1.32E−02
Mucin type O-glycan biosynthesis	1.33E−02
Thyroid hormone synthesis	1.51E−02
EGFR tyrosine kinase inhibitor resistance	1.58E−02

Endocrine resistance	1.69E−02
Inflammatory mediator regulation of TRP channels	1.73E−02
DNA replication	1.73E−02
Human immunodeficiency virus 1 infection	1.75E−02
GABAergic synapse	1.75E−02
Salivary secretion	1.81E−02
Apelin signaling pathway	1.88E−02
Chronic myeloid leukemia	1.91E−02
Cortisol synthesis and secretion	1.94E−02
Melanogenesis	1.99E−02
Gastric acid secretion	1.99E−02
Pancreatic cancer	1.99E−02
Adherens junction	2.10E−02
One carbon pool by folate	2.10E−02
2-Oxocarboxylic acid metabolism	2.10E−02
Basal cell carcinoma	2.10E−02
Estrogen signaling pathway	2.14E−02
Fatty acid degradation	2.16E−02
Antifolate resistance	2.16E−02
Calcium signaling pathway	2.32E−02
Hippo signaling pathway	2.48E−02
Antigen processing and presentation	2.49E−02
Gastric cancer	2.52E−02
Description	q-value
Cytokine-cytokine receptor interaction	7.43E−21
Viral protein interaction with cytokine and cytokine receptor	2.92E−14
Cell adhesion molecules	6.10E−13
PI3K-Akt signaling pathway	2.90E−12
Hematopoietic cell lineage	5.95E−11
Chemokine signaling pathway	1.49E−09
Dilated cardiomyopathy	1.81E−09
ECM-receptor interaction	2.37E−09
Osteoclast differentiation	5.60E−09
Hypertrophic cardiomyopathy	1.57E−08
TNF signaling pathway	2.37E−08
MAPK signaling pathway	2.74E−08
Arrhythmogenic right ventricular cardiomyopathy	2.90E−08
Toxoplasmosis	6.04E−08
JAK-STAT signaling pathway	7.69E−08
Amoebiasis	1.05E−07
Complement and coagulation cascades	1.46E−07
Epstein-Barr virus infection	1.82E−07
Human papillomavirus infection	1.86E−07
Focal adhesion	2.93E−07
Rap1 signaling pathway	3.17E−07
Platelet activation	4.43E−07
Human T-cell leukemia virus 1 infection	1.61E−06
NF-kappa B signaling pathway	1.61E−06
Malaria	1.99E−06
Leishmaniasis	2.09E−06

Measles	2.53E−06
Small cell lung cancer	3.69E−06
AGE-RAGE signaling pathway in diabetic complications	3.93E−06
Th17 cell differentiation	3.93E−06
Chagas disease	3.93E−06
Coronavirus disease - COVID-19	7.08E−06
Kaposi sarcoma-associated herpesvirus infection	8.51E−06
Th1 and Th2 cell differentiation	8.51E−06
Ras signaling pathway	1.21E−05
Pertussis	1.21E−05
Viral myocarditis	1.49E−05
Tuberculosis	1.49E−05
Protein digestion and absorption	1.63E−05
Rheumatoid arthritis	2.00E−05
Legionellosis	2.11E−05
Inflammatory bowel disease	2.32E−05
Valine, leucine and isoleucine degradation	2.80E−05
Phagosome	3.75E−05
Human cytomegalovirus infection	3.75E−05
Relaxin signaling pathway	4.06E−05
Yersinia infection	4.82E−05
Salmonella infection	5.62E−05
Leukocyte transendothelial migration	5.62E−05
PD-L1 expression and PD-1 checkpoint pathway in cancer	1.17E−04
Parathyroid hormone synthesis, secretion and action	1.41E−04
Influenza A	1.41E−04
Toll-like receptor signaling pathway	1.48E−04
Primary immunodeficiency	1.60E−04
Glyoxylate and dicarboxylate metabolism	1.70E−04
C-type lectin receptor signaling pathway	2.06E−04
Circadian entrainment	2.39E−04
Transcriptional misregulation in cancer	2.81E−04
Gap junction	3.37E−04
Glycosaminoglycan biosynthesis - chondroitin sulfate / dermatan sulfate	3.49E−04
Axon guidance	3.49E−04
T cell receptor signaling pathway	4.68E−04
Tryptophan metabolism	5.49E−04
Hepatitis B	5.61E−04
Hepatitis C	6.71E−04
Lipid and atherosclerosis	6.80E−04
Apoptosis	8.34E−04
Proteoglycans in cancer	8.34E−04
Intestinal immune network for IgA production	9.20E−04
NOD-like receptor signaling pathway	9.25E−04
cAMP signaling pathway	9.52E−04
Cysteine and methionine metabolism	9.77E−04
Regulation of actin cytoskeleton	1.09E−03
Oxytocin signaling pathway	1.18E−03
Prostate cancer	1.18E−03

Staphylococcus aureus infection	1.22E−03
Glycine, serine and threonine metabolism	1.62E−03
Lysine degradation	1.78E−03
Adrenergic signaling in cardiomyocytes	1.96E−03
Fc gamma R-mediated phagocytosis	1.96E−03
Alcoholic liver disease	2.09E−03
Natural killer cell mediated cytotoxicity	2.26E−03
B cell receptor signaling pathway	2.53E−03
Amphetamine addiction	2.82E−03
Viral carcinogenesis	3.18E−03
Cocaine addiction	3.18E−03
Breast cancer	3.22E−03
Cell cycle	5.23E−03
Hepatocellular carcinoma	5.23E−03
Glutamatergic synapse	5.69E−03
Notch signaling pathway	6.45E−03
Insulin secretion	6.87E−03
Cellular senescence	7.09E−03
Phospholipase D signaling pathway	7.17E−03
Morphine addiction	7.18E−03
Cholinergic synapse	8.19E−03
Glioma	1.04E−02
Vascular smooth muscle contraction	1.08E−02
IL-17 signaling pathway	1.09E−02
cGMP-PKG signaling pathway	1.09E−02
Acute myeloid leukemia	1.13E−02
ABC transporters	1.15E−02
Cushing syndrome	1.25E−02
Fluid shear stress and atherosclerosis	1.25E−02
Other types of O-glycan biosynthesis	1.26E−02
Colorectal cancer	1.32E−02
Mucin type O-glycan biosynthesis	1.33E−02
Thyroid hormone synthesis	1.51E−02
EGFR tyrosine kinase inhibitor resistance	1.58E−02
Endocrine resistance	1.69E−02
Inflammatory mediator regulation of TRP channels	1.73E−02
DNA replication	1.73E−02
Human immunodeficiency virus 1 infection	1.75E−02
GABAergic synapse	1.75E−02
Salivary secretion	1.81E−02
Apelin signaling pathway	1.88E−02
Chronic myeloid leukemia	1.91E−02
Cortisol synthesis and secretion	1.94E−02
Melanogenesis	1.99E−02
Gastric acid secretion	1.99E−02
Pancreatic cancer	1.99E−02
Adherens junction	2.10E−02
One carbon pool by folate	2.10E−02
2-Oxocarboxylic acid metabolism	2.10E−02
Basal cell carcinoma	2.10E−02

Estrogen signaling pathway	2.14E−02
Fatty acid degradation	2.16E−02
Antifolate resistance	2.16E−02
Calcium signaling pathway	2.32E−02
Hippo signaling pathway	2.48E−02
Antigen processing and presentation	2.49E−02
Gastric cancer	2.52E−02

Table S3. Thirty-four differential metabolites identified in positive and negative mode.

In positive mode

NO	$[M+H]^+$ (m/z)	Delta (ppm)	Formula	Metabolites	VIP	P
1	99.0650	−9.33	$C_5H_{10}NO$	2-Piperidinone	1.7567385022	0.0128659156
2	101.1195	−9.43	$C_6H_{16}N$	Hexylamine	2.0379096022	0.0023337061
3	111.0427	−4.94	$C_4H_6N_3O$	Cytosine	2.0418150497	0.0010972827
4	121.0883	−7.34	$C_8H_{12}N$	Phenylethylamine	1.9814597721	0.0024521583
5	160.0991	−6.23	$C_{10}H_{13}N_2$	Anatabine	1.7979543685	0.0053108897
6	162.0671	−6.28	$C_{10}H_{11}O_2$	4,5-Dihydro-1-benzoxepin-3(2H)-one	2.3499237288	0.0000092619
7	188.1285	6.10	$C_7H_{17}N_4O_2$	L-Targinine	1.6074156932	0.0156388593
8	190.0366	−6.65	$C_9H_7N_2O_3$	Nitroxoline	1.2314601400	0.0471657634
9	215.0550	−3.86	$C_5H_{15}NO_6P$	Glycerylphosphorylethanolamine	1.6064902294	0.0165914871
10	220.1089	−4.70	$C_{13}H_{17}O_3$	1-(5-Acetyl-2-hydroxyphenyl)-3-methyl-1-butanone	2.3521427735	0.0000073518
11	293.1979	−3.96	$C_{17}H_{28}NO_3$	Nonivamide	2.4139510116	0.0000004355
12	336.2651	−3.91	$C_{21}H_{37}O_3$	Pregnanetriol	1.6838470507	0.0054941678
13	413.3291	−0.56	$C_{27}H_{44}NO_2$	5alpha-Tomatidan-3-one	1.5118790876	0.0139911382
14	505.3514	−3.65	$C_{26}H_{53}NO_6P$	LysoPC(P-18:1(9Z))	1.9615013500	0.0004286134
15	511.4947	−3.32	$C_{32}H_{66}NO_3$	Cer(d18:0/14:0)	1.3676883747	0.0433282614
16	545.3443	−7.11	$C_{28}H_{53}NO_7P$	LysoPC(20:3(5Z,8Z,11Z))	1.8926985265	0.0011333993
17	723.5180	−3.16	$C_{41}H_{75}NO_7P$	PE(18:3(6Z,9Z,12Z)/P-18:1(11Z))	2.3330819395	0.0000051317
18	728.5802	−4.09	$C_{41}H_{82}N_2O_6P$	SM(d18:1/18:1(11Z))	1.4632555185	0.0264426424
19	743.5798	−4.12	$C_{42}H_{83}NO_7P$	PC(16:0/P-18:1(11Z))	1.5551483320	0.0170014236
20	771.5752	−3.44	$C_{43}H_{83}NO_8P$	PC(15:0/20:2(11Z,14Z))	1.9614160117	0.0008837868

In negative mode

NO	$[M-H]^-$ (m/z)	Delta (ppm)	Formula	Metabolites	VIP	P
1	774.5362	−6.28	$C_{42}H_{78}O_{10}P$	PG(18:0/18:2(9Z,12Z))	1.9623653742	0.0009273760
2	282.2534	−8.81	$C_{18}H_{33}O_2$	(Z)-13-Octadecenoic acid	1.9108915324	0.0010083556
3	792.4869	−9.14	$C_{44}H_{72}O_{10}P$	PG(16:1(9Z)/22:6 (4Z,7Z,10Z,13Z,16Z,19Z))	2.0001715309	0.0010713927
4	912.5654	−8.14	$C_{49}H_{84}O_{13}P$	PI(18:0/22:5(4Z,7Z,10Z,13Z,16Z))	2.0956677860	0.0002078235
5	796.5192	−7.89	$C_{44}H_{75}O_{10}P$	PG(16:0/22:5(4Z,7Z,10Z,13Z,16Z))	1.4884584386	0.0174221265
6	842.5034	0.77	$C_{44}H_{73}O_{15}$	Vinaginsenoside R1	1.5139760286	0.0193148061
7	816.4863	−9.61	$C_{46}H_{72}O_{10}P$	PG(18:3(6Z,9Z,12Z)/22:6 (4Z,7Z,10Z,13Z,16Z,19Z))	1.7761854290	0.0031726938
8	228.0051	−7.22	$C_{13}H_7S_2$	5-Ethynyl-5'-(1-propynyl)-2,2'-bithiophene	1.2755898001	0.0495846592
9	304.2378	−8.00	$C_{20}H_{31}O_2$	Mesterolone	1.3987605779	0.0261059783
10	174.0170	3.40	$C_6H_5O_6$	cis-Aconitic acid	1.4947798644	0.0234651146
11	213.0073	0.71	$C_5H_8Cl_2N_3O_2$	Carmustine	1.4687272903	0.0179947660
12	818.5037	−7.38	$C_{46}H_{74}O_{10}P$	PG(18:2(9Z,12Z)/22:6 (4Z,7Z,10Z,13Z,16Z,19Z))	1.5661563684	0.0119455349
13	111.0427	−4.94	$C_2H_4NO_2$	Glycine	1.4715910009	0.0206321526
14	256.2379	−8.97	$C_{16}H_{31}O_2$	Isopalmitic acid	1.4663757188	0.0240594117
